# Determination of pyrroloquinoline quinone by enzymatic and LC-MS/MS methods to clarify its levels in foods

**DOI:** 10.1371/journal.pone.0209700

**Published:** 2018-12-21

**Authors:** Chikara Kato, Emiko Kawai, Naoki Shimizu, Tsuyoshi Mikekado, Fumiko Kimura, Teruo Miyazawa, Kiyotaka Nakagawa

**Affiliations:** 1 Food and Biodynamic Chemistry Laboratory, Graduate School of Agricultural Science, Tohoku University, Sendai, Japan; 2 Niigata Research Laboratory, Mitsubishi Gas Chemical Company, Inc., Niigata, Japan; 3 Department of Human Health and Nutrition, Shokei Gakuin University, Natori, Japan; 4 New Industry Creation Hatchery Center (NICHe), Tohoku University, Sendai, Japan; 5 Food and Health Science Research Unit, Graduate School of Agricultural Science, Tohoku University, Sendai, Japan; University of Missouri Columbia, UNITED STATES

## Abstract

Pyrroloquinoline quinone (PQQ) is believed to be a new B vitamin-like compound, and PQQ supplementation has received attention as a possible treatment for diseases including dementia and diabetes. However, the distribution of PQQ in foods is unclear, due to the difficulty in analyzing the compound. Therefore, in this study, enzymatic and LC-MS/MS methods were optimized to enable an accurate analysis of PQQ in foods. The optimized methods were applied to the screening of foods, in which PQQ contents were identified in ng/g or ng/mL levels. Furthermore, we newly found that some foods related to acetic acid bacteria contain PQQ at 1.94~5.59 ng/mL higher than beer, which is known to contain relatively high amounts of PQQ. These results suggest that the optimized methods are effective for the screening of foods containing PQQ. Such foods with high PQQ content may be valuable as functional foods effective towards the treatment of certain diseases.

## Introduction

**Fig 1:**
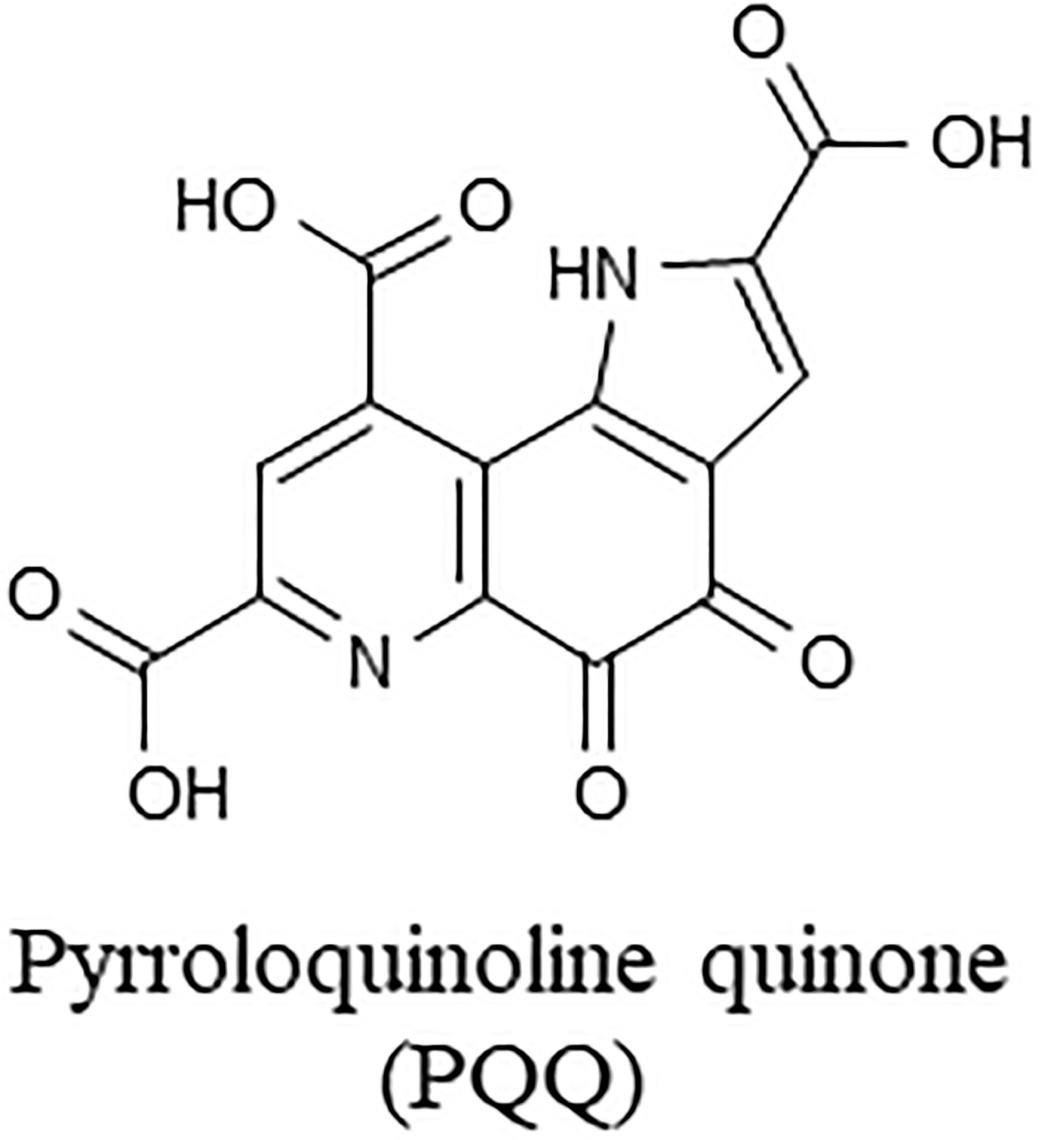
Structure of pyrroloquinoline quinone (PQQ).

A water soluble quinone, pyrroloquinoline quinone (PQQ) (Fig 1), was first identified as an enzymatic cofactor in bacteria in 1979 [1]. After vitamin B2 and niacin, PQQ is the third compound that acts as a coenzyme of oxidoreductase such as alcohol dehydrogenase [1] and glucose dehydrogenase (GDH) [1–4]. Subsequently, symptoms caused by PQQ deficiency [5] and the coenzyme functions of PQQ [6, 7] were discovered in mouse studies. Based on these results, PQQ has been considered as a new B vitamin like compound [5–8]. More recent studies have reported additional activities (e.g. antioxidative and neuroprotective effects) of PQQ [9–12], and therefore, PQQ supplementation has received attention as a possible treatment for certain diseases including dementia and diabetes [13–16].

The beneficial effects of PQQ may be associated with the presence of PQQ (at trace levels) in human tissues and body fluids [17–21]. Because eukaryotic cells cannot synthesize PQQ [22], foods have been said to be the only source of PQQ for humans [23, 24]. However, the distribution of PQQ in foods is still unclear, mainly due to the difficulty in analyzing the compound.

So far, enzymatic and mass spectrometric (MS) methods have been applied to PQQ analysis [17–21, 23–26]. In the enzymatic method, PQQ is quantified through enzyme (*Escherichia coli* GDH) activity measurement [20, 21, 25, 26]. The enzymatic method has been utilized to evaluate the absorption of PQQ into blood and tissues after oral administration to rats [20] and humans [21]. However, the selectivity and accuracy of the method (i.e. whether the method can discriminate PQQ from various PQQ derivatives) are still unclear, and to the best of our knowledge, the method has never been applied for the analysis of PQQ in foods and raw materials.

Regarding MS methods, Kumazawa et al. utilized gas chromatography (GC)-MS with selected ion monitoring mode, and confirmed the presence of trace levels of PQQ in human and rat samples [17, 18]. Also, Noji et al. applied liquid chromatography-tandem MS (LC-MS/MS) with multiple reaction monitoring (MRM) and analyzed PQQ and PQQ derivatives in foods [24]. However, the quantitative values of PQQ were considerably different between the GC-MS and LC-MS/MS methods [23, 24].

In this study, to address the aforementioned analytical concerns, several parameters of both enzymatic and MS methods were optimized, and extraction conditions of PQQ from food samples were also investigated. Upon these investigations, the PQQ contents in medium determined by the enzymatic method well agreed with that of the LC-MS/MS method, suggesting that the developed methods are useful for the screening of PQQ in foods and raw materials. The results of this study may be valuable for the application of PQQ for nutraceutical purposes.

## Materials and methods

### Materials

Pyrroloquinoline quinone disodium salt (PQQ2Na, commercially known as BioPQQ) and isotope labeled PQQ2Na (^13^C-PQQ2Na) were provided by Mitsubishi Gas Chemical Company, Inc. (Niigata, Japan). ^13^C-PQQ2Na consisted of three stable isotopes; ^13^C_14_H_6_N_2_O_8_ ([U-^13^]C-PQQ), C^13^C_13_H_6_N_2_O_8_, and C_2_^13^C_12_H_6_N_2_O_8_ were present in the ratio of 70:25:4 (mol%). PQQ2Na and ^13^C-PQQ2Na were individually dissolved in 20 mM ammonium acetate aqueous solution to prepare reference standards. GDH (PQQ-dependent; from microorganism, EC1.1.5.2) was purchased from TOYOBO ENZYME (Düsseldorf, Germany) [27]. Other chemicals were of the highest purity commercially available.

### Samples

A methanol-utilizing bacterium (*Hyphomicrobium* sp. strain TK 0441), known to produce PQQ [28,], was cultured in a suitable medium containing 1 μg/mL Fe, 150 μg/mL Mg, and trace elements in an Erlenmeyer flask at 30°C with shaking (220 rpm) for 14 days [29]. The culture broth was centrifuged at 10,000 *g* for 10 min, and the supernatant was collected. The supernatant (35 μL) was diluted with 965 μL water and subjected to PQQ extraction as described below.

Foods previously analyzed by Kumazawa et al. and/or Noji et al. [23, 24] and 8 kinds of vinegar (A–H) were purchased at a local supermarket in Sendai, Japan. Vinegar A was purchased at KALDI COFFEE FARM Sendai store (Sendai, Japan) and Vinegar F was purchased at SEIJO ISHII S-pal Sendai store (Sendai, Japan). Other foods were purchased at Miyagi Consumer’s Cooperative Society Kimachi store (Sendai, Japan). Green tea, oolong tea, beer, and 8 kinds of vinegar were directly subjected to extraction. Other foods (fermented soybeans (natto), green pepper, spinach, tomato, parsley, and rape blossoms) were freeze-dried followed by powderization. The powder (2–5 g) was suspended in 20 mL water, vortexed for 5 min, and sonicated for 30 min. After centrifugation at 1,500 *g* at 4°C, for 15 min, the supernatants were subjected to PQQ extraction [24].

### PQQ extraction

The medium sample (1 mL) was mixed with or without 100 μL of internal standard (10 μM ^13^C-PQQ2Na) in a polypropylene tube. Similarly, each food sample (1 mL) was mixed with or without 10 μL of 1 μM ^13^C-PQQ2Na. PQQ was extracted based on previous methods with modification [17, 24]. To the samples, 3 mL of ethyl acetate was added. This mixture was vortexed for 5 min, centrifuged at 1,500 *g* at 4°C for 15 min, and the supernatant was removed for delipidation. The lower water layer was mixed with 250 μL of 6 M HCl and 5 mL of ethyl acetate. This mixture was vortexed for 5 min, centrifuged at 1,500 *g* at 4°C for 15 min, and the upper ethyl acetate layer (containing PQQ) was collected. The collected upper layer was mixed with 500 μL of water, followed by evaporation under nitrogen gas. The remaining water layer was mixed with 100 μL of 6 M HCl, and subjected to an Oasis HLB cartridge (Waters, Milford, MA, USA) equilibrated with 1 mM HCl. PQQ was eluted with 500 μL of 0.5% (v/v) pyridine/water, and the eluate was evaporated under nitrogen gas. The medium and food extracts were dissolved in 1 mL and 100 μL of 20 mM ammonium acetate, respectively.

### Enzymatic method

PQQ was measured by using previously described GDH-based enzymatic methods with modifications [25, 26]. GDH (1 mg) was dissolved in 1 mL of 0.1 M phosphate buffer (containing 1 mM ethylenediamine-N,N,N',N'-tetraacetic acid (EDTA) and 2 M KBr; pH 7.3). For inactivation of GDH, the solution was dialyzed against the buffer at 4°C for 30 h to remove GDH-bound PQQ [2, 30, 31]. The obtained apo-GDH was dialyzed against 20 mM 3-morpholinopropanesulfonic acid (MOPS) buffer (pH 7.0) at 4°C for 30 h. The protein concentration of this solution was measured, and the solution was diluted with 20 mM MOPS buffer (containing 0.1% Triton X-100, 1 mM CaCl_2_, and 0.1% bovine serum albumin) to a concentration of 0.4 ng/mL. The apo-GDH solution (250 μL) was incubated with either 25 μL of sample extracts or PQQ standards (0.25–1.5 ng/mL) at 25°C for 30 min to reactivate GDH [31, 32]. Distilled water was used as a blank. The resultant holo-GDH solution (40 μL) was mixed with 627 μL of 20 mM MOPS buffer (containing 2 mM CaCl_2_, 400 μM phenazine methosulfate, and 200 μM 2, 6-dichloroindophenol (DCIP) sodium salt). After adding 73 μL of 1.2 M glucose aqueous solution, the GDH activity was determined by measuring the rate of discoloration of DCIP blue at 600 nm with Infinite 200 PRO (Tecan Japan Co., Ltd., Kanagawa, Japan), and ΔABS was calculated by (ABS _1 min, sample_ − ABS _21 min, sample_) − (ABS _1 min, blank_ − ABS _21 min, blank_). Concentrations of PQQ in sample extracts were calculated using the equation corresponding to the external standard curve of ΔABS and were adjusted by the percentage recovery. The percentage recovery was calculated by [(C_p_-C_0_) / I_p_]×100, where C_p_ is concentration of sample extract added with internal standard, C_0_ is concentration of sample extract added without internal standard, and I_p_ is internal standard concentration.

### LC-MS/MS method

PQQ was determined by LC-MS/MS based on our previous method with slight modifications [33]. The LC system consisted of Shimadzu LC-20AD pumps (Shimadzu, Kyoto, Japan), Shimadzu CTO-20A column oven (Shimadzu, Kyoto, Japan) and a Shimadzu SIL-20AC autosampler (Shimadzu, Kyoto, Japan) having a 100 μL sample loop. An ODS column (Atlantis T3 column, 3 μm, 2.1×100 mm, Waters, MA, USA) was used with a binary gradient consisting of solvent A (water containing 10 mM dibutylammonium acetate) and solvent B (acetonitrile). The gradient profile was as follows: 0–16 min, 0–60% B linear; 16–16.1 min, 60–100% B linear; 16.1–20 min, 100% B, 20–20.1 min, 100–0% B linear; 20.1–33 min, 0% B. The flow rate was 0.2 mL/min, and the column temperature was maintained at 40°C. The column eluent was sent to a hybrid triple quadrupole/liner ion-trapped tandem mass spectrometer (4000QTRAP, SCIEX, Tokyo, Japan). Product ion scan and optimization of MS/MS parameters were conducted with PQQ2Na and ^13^C-PQQ2Na standards under electro-spray ionization (ESI) (negative). Samples (1 μL for medium extract and 10 μL for food extracts) or PQQ standards (10 μL; 15–650 pg on column) were injected and PQQ was determined using MRM transitions as follows: PQQ, *m/z* 329 > 241; ^13^C-PQQ, *m/z* 343 > 253. Concentrations of PQQ in sample extracts were calculated using the equation corresponding to the external standard curve and were adjusted by the percentage recovery of the added ^13^C-PQQ (the internal standard).

## Results and discussion

Foods have been said to be the only source of PQQ for humans [23, 24], however, the distribution of PQQ in foods is still unclear, mainly due to the difficulty in analyzing the compound. We therefore aimed to quantify PQQ by a GDH-based enzymatic method and by LC-MS/MS. We tried to confirm the actual concentration of PQQ in foods, which may help clarify the natural distribution of PQQ.

### Enzymatic analysis of PQQ

**Fig 2:**
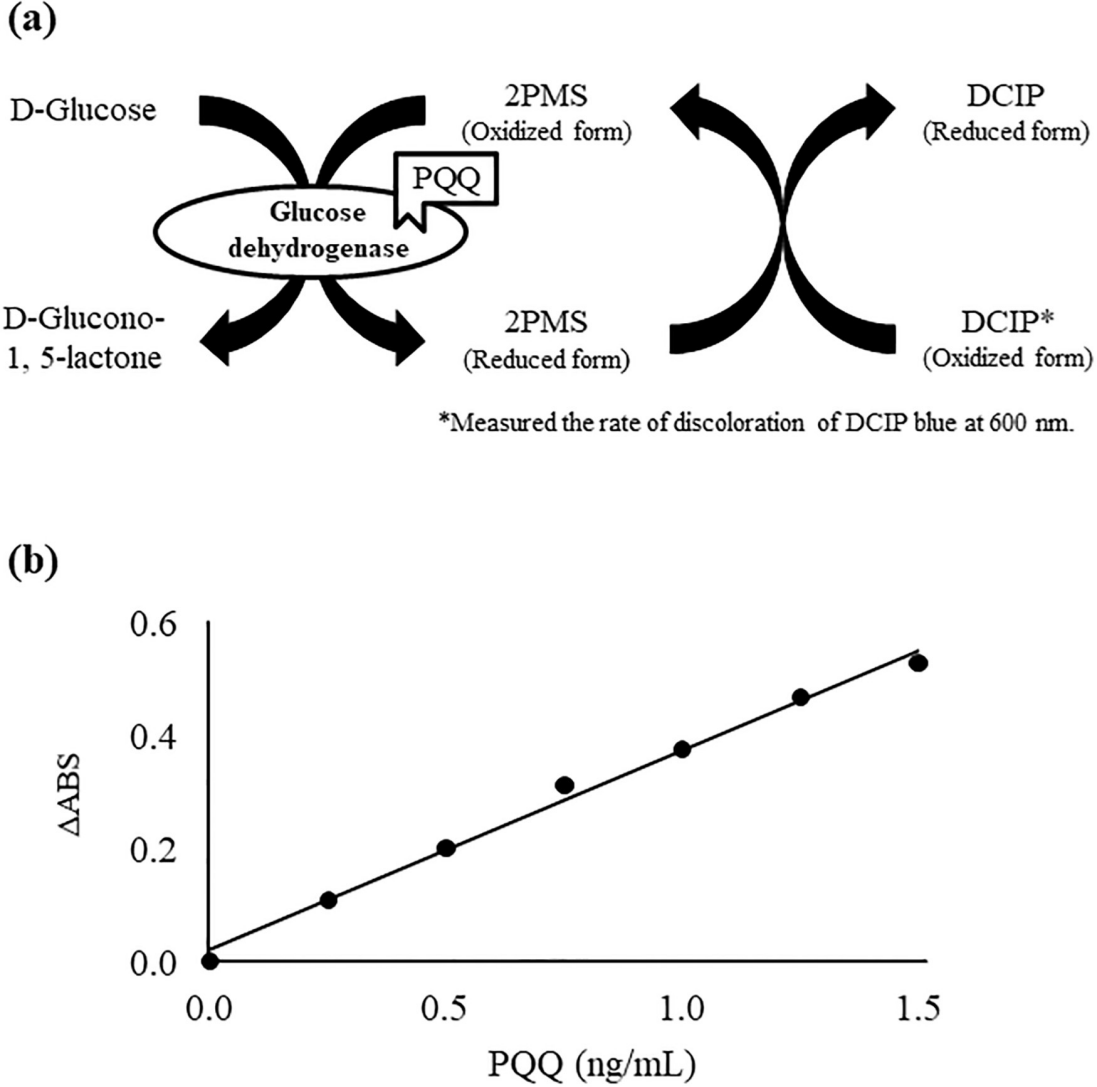
(a) The measurement mechanism of enzymatic activity. The GDH activity was determined by measuring the rate of discoloration of DCIP blue at 600 nm with Infinite 200 PRO, and ΔABS was calculated by (ABS _1 min, sample_ − ABS _21 min, sample_) − (ABS _1 min, blank_ − ABS _21 min, blank_). (b) Standard curve for PQQ determination by the enzymatic method. The reconstitution mixture contained 20 mM MOPS buffer, pH 7.0, 0.1% TritonX-100, 1 mM CaCl2, 0.1% bovine serum albumin, variable concentrations of PQQ, and 0.4 ng/mL GDH. The incubation period was 30 min. Detailed analytical procedures are described in Materials and methods section.

Analysis of PQQ via enzymatic methods have been attempted in previous studies [25–27]. By measuring the activity of GDH in its active holo-form (i.e. PQQ bound GDH) (Fig 2a), Ameyama et al. tried to identify the role of PQQ as a coenzyme [4]. Geiger et al. made improvements of the method to enable a more stable analysis of PQQ [26], and more recently, Rucker et al. quantitatively analyzed PQQ contents in human plasma with the use of enzymatic methods [20, 21]. We therefore aimed to analyze PQQ in food materials with the use of the enzymatic method, but stable analysis of PQQ was difficult presumably due to the contamination of different PQQ enzymes during the preparation of GDH that caused a decrease in enzyme activity [25, 26]. Also, since previous methods required the cultivation of bacteria and the purification of enzymes from the bacterial membrane which were time-consuming [25, 26], we considered that it was necessary to simplify the method if we were to conduct a screening of foods containing PQQ.

To overcome such problems, we utilized a commercial GDH enzyme [27] with high activity levels for the analysis of PQQ in foods [2, 25]. With regard to GDH enzymes, GDH can be classified into two types, membrane-bound GDH (mGDH) and soluble GDH (sGDH), which possess distinct structures and PQQ binding sites [28, 32, 34, 35]. The GDH utilized in previous studies were isolated from bacterial membranes, and thus were considered to be mGDH [25, 26]. However, compared to mGDH, sGDH demonstrates a higher affinity to PQQ, and quickly binds to PQQ [34]. We thus considered that the use of a commercially available sGDH will allow for higher enzyme activity as well as quicker holo-GDH reconstitution than conventional methods. To further enhance the above benefit, we also purified the commercial sGDH by removing metal ions via EDTA treatment [31] and by dialysis to obtain a purified apo-GDH. With the use of this apo-GDH, we evaluated PQQ analysis conditions. Consequently, the required enzyme concentration for PQQ analysis was reduced to 0.4 ng/mL, compared to previous methods where the required enzyme concentration was 0.25 mg/mL. Moreover, the reconstitution time of the holo-enzyme to enable a stable PQQ-concentration dependent GDH activity was 30 min (conventional reconstitution times were 1–2 h). As a result of the above measures, we were able to establish a sensitive and convenient enzymatic method that can produce high-precision standard curve with correlation coefficient over 0.99 in the range of 0.25–1.5 ng/mL (Fig 2b). Limit of detection (LOD) and limit of quantitation (LOQ) were 0.25 and 0.1 ng/mL, respectively.

### LC-MS/MS analysis of PQQ

Along with the enzymatic method, analysis of PQQ has previously been pursued with MS methods [18, 19, 23, 24]. Kumazawa et al. quantified PQQ in foods such as fermented soybeans (natto) and green tea by GC-MS [23], and reported that the PQQ levels in these foods were in the range of 9.2–61.0 ng/g fresh weight or ng/mL, whereas with the use of LC-MS/MS, Noji et al. reported that the PQQ levels in these foods range from 0.19–7.02 ng/g fresh weight or ng/mL [24]. Because these values differed greatly, we therefore aimed to confirm the actual concentrations of PQQ in such foods with the use of LC-MS/MS with no derivatization. With regard to the LC-MS/MS analysis of PQQ, previous methods have demonstrated certain limitations such as low retention of PQQ on the column (Rt 2.2 min) [24]. Moreover, since the detection limits were about 50 pg [24], we considered that it was necessary to construct a method to analyze PQQ with high accuracy and sensitivity.

**Fig 3:**
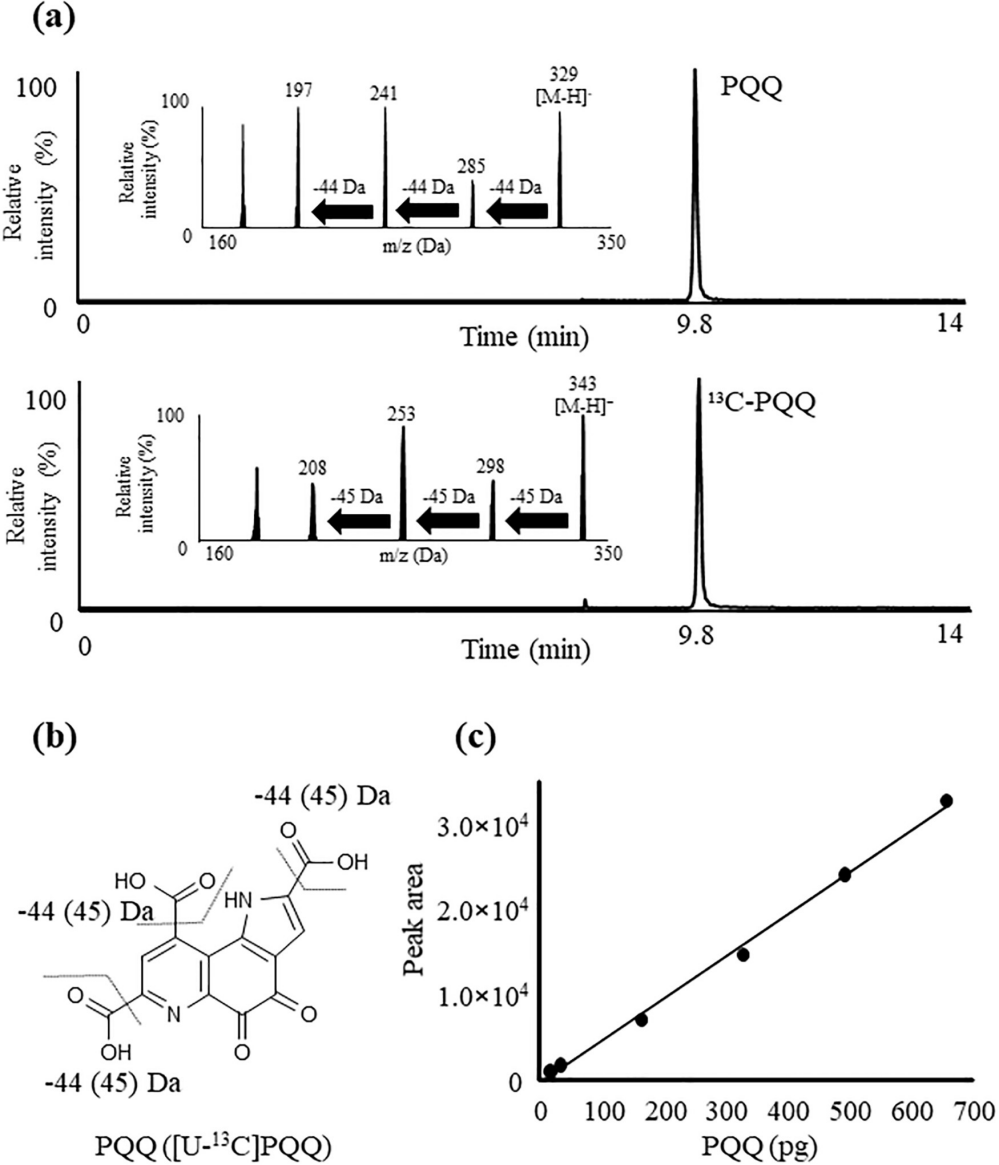
(a) Product ion mass spectrum and ESI chromatograms of PQQ and ^13^C-PQQ. PQQ and ^13^C-PQQ standards (10 μM in 30% (v/v) acetonitrile/10 mM DBAA aqueous solution) were each infused directly into the MS/MS apparatus at a flow rate of 5 μL/min. A mixture of standards (2 pmol each) was analyzed by LC-MS/MS. (b) The proposed fragmentation pattern of PQQ and [U-^13^]C-PQQ. (c) Calibration curve of PQQ by LC-MS/MS. Different amounts of PQQ (15–650 ng) were analyzed in the ESI mode. Detailed analytical procedures are described in Materials and methods section.

The main difficulty in analyzing PQQ with LC-MS/MS is the fact that the structure and electric charge of PQQ are significantly affected by pH [36], due to the presence of three carboxyl groups as well as pyridine and pyrrole rings in the chemical structure of PQQ. To overcome such difficulties, we previously applied dibutylammonium acetate, an ion-pairing reagent, to construct an isocratic LC-MS/MS method that enables the sufficient retention of PQQ on the column [34]. Based on this method, in this study, we utilized a gradient flow using acetonitrile which strongly elutes PQQ. As a result, PQQ was retained on the column (Rt 9.8 min) and chromatograms demonstrated sharp peaks of PQQ. Also, consistent with our previous reports, the precursor ion of PQQ (*m/z* 329 [M-H]^-^) provided the product ions of *m/z* 285 [M-H-CO_2_]^-^, *m/z* 241 [M-H-2CO_2_]^-^, *m/z* 197 [M-H-3CO_2_]^-^, and the precursor ion of [U-^13^]C-PQQ (*m/z* 343 [M-H]^-^) provided the product ions of *m/z* 298 [M-H-^13^CO_2_]^-^, *m/z* 253 [M-H-2^13^CO_2_]^-^, *m/z* 208 [M-H-^13^CO_2_]. With the use of MRM pairs (PQQ, *m/z* 329 > 241, ^13^C-PQQ, *m/z* 343 > 253) based on product ion analysis (Fig 3a and 3b), we were able to construct a LC-MS/MS method that can produce standard curve with correlation coefficient over 0.99 in the concentration range of 15–6,000 pg (Fig 3c). LOD and LOQ were 1.5 ng/mL (15 pg on column) and 0.5 ng/mL (5 pg on column), respectively.

### PQQ extraction

As described above, studies on the analysis of PQQ in foods have been limited to only few studies [23, 24], where the extraction of PQQ from foods have mainly been conducted in the following two ways [17, 24]. Suzuki et al. extracted PQQ from foods using liquid-liquid extraction with n-butanol followed by a reversed phase solid-phase extraction [17]. Since the extraction method required many processes, Noji et al. constructed a simpler liquid-liquid extraction method using ethyl acetate [24]. However, because this extraction method did not apply any solid-phase extraction procedures, there were risks of matrix compounds interfering with MS analysis. Therefore, we combined the liquid-liquid extraction method using ethyl acetate introduced by Noji et al. [24] with the solid-phase extraction described by Suzuki et al. [17] and improved certain aspects of the extraction method.

Since the electric charge of PQQ is significantly affected by pH [36], the extraction with ethyl acetate, water, and HCl highly affects the recovery of PQQ. We thus evaluated the recovery of PQQ to the ethyl acetate phase when the pH of the water phase was set between 0.5–4.0 using HCl. As a result, between pH 3–4, PQQ mostly retained on the water phase, while at lower pH, PQQ was mostly transferred to the ethyl acetate phase. Therefore, we decided to first remove lipids from the sample under neutral conditions, and then recover PQQ to the ethyl acetate layer under strong acidic conditions.

With regard to solid-phase extraction, we used the Oasis HLB cartridge which can be used in a wide range of pH, and has a unique hydrophile-lipophile balance. As a result, we were able to efficiently recover PQQ even when applying samples under acidic conditions. A recovery test using PQQ2Na and/or ^13^C-PQQ2Na confirmed that the aforementioned extraction method can recover 70–80% of each standard.

### Determination of PQQ from medium and foods by the enzymatic and LC-MS/MS methods

**Fig 4:**
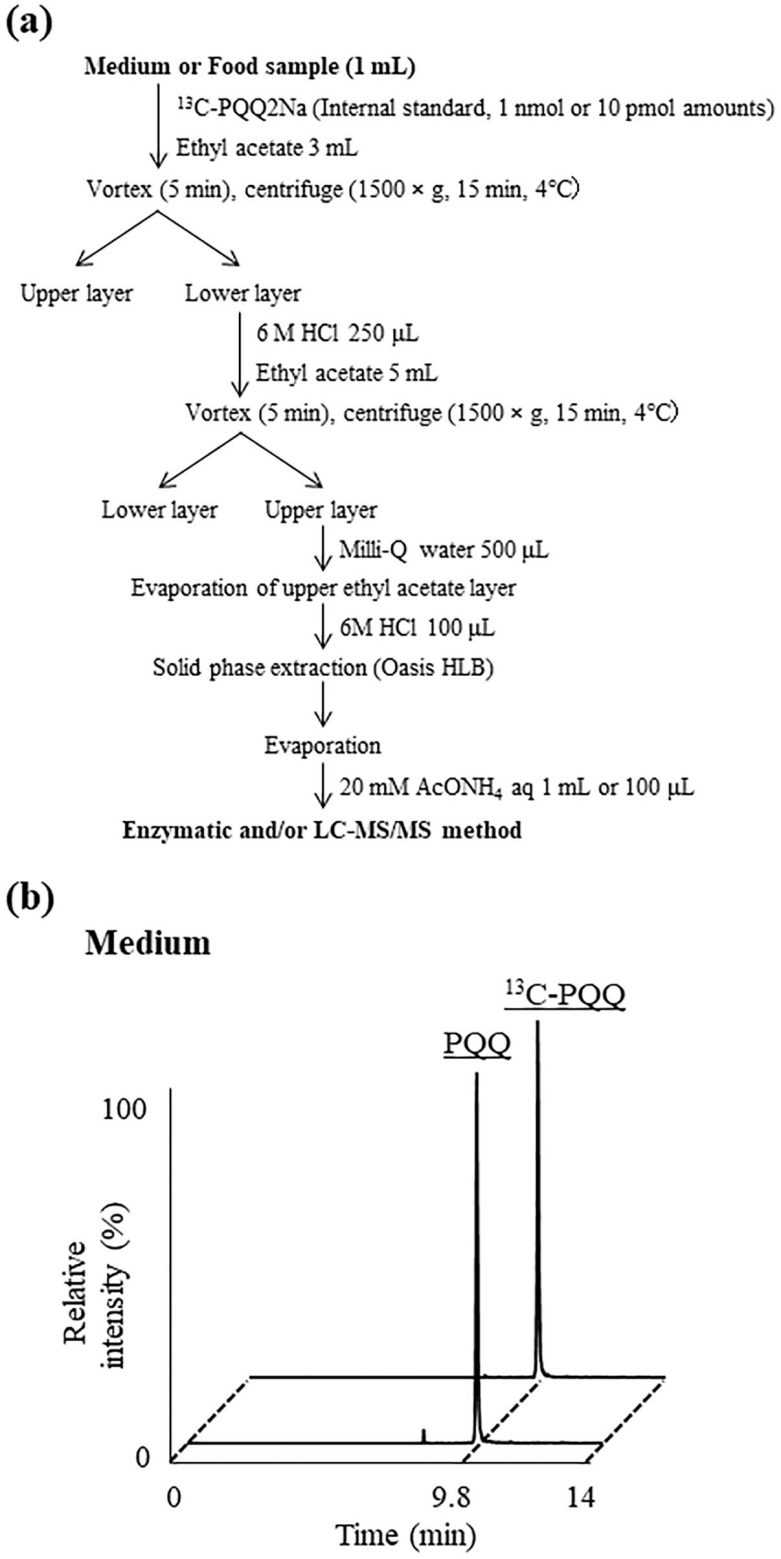
(a) The scheme of PQQ extraction. (b) Chromatogram peaks of PQQ and ^13^C-PQQ (internal standard) from medium extraction. Detailed analytical procedures are described in Materials and methods section.

In order to evaluate the selectivity and accuracy of the enzymatic method and LC-MS/MS method, we analyzed PQQ contents in medium that was known to definitely contain PQQ (detailed information of the sample was described in Samples section) [29] via each method (Fig 4a). The PQQ contents in the medium were determined as 11.2 ± 2.12 μg/mL by the enzymatic method, and 10.4 ± 0.37 μg/mL by the LC-MS/MS method (Fig 4b). The fact that the measured values were close suggests the reliability of each method. Due to the high reactivity of PQQ with amino acids [19, 37, 38], there were concerns of PQQ reacting with amino acids contained in the medium and the resultant PQQ derivatives being detected by the enzymatic method. However, since both values were similar, we considered that the enzymatic method allows for the selective detection of only PQQ. Additionally, the recovery rates were 74.1% in the enzymatic method and 79.2% in the LC-MS/MS method, suggesting the high accuracy of each method. Given the above results, we considered that the optimized extraction method as well as the enzymatic and LC-MS/MS methods could be applied to quantify the amounts of PQQ in various samples.

We therefore applied the more convenient enzymatic method to the screening of foods that were previously reported to contain PQQ (Fig 4a) [23, 24]. Consistent with previous studies, it was confirmed that the PQQ level in foods was ng level (Table 1). The fact that the PQQ contents in certain foods were either trace amounts or below detection limits may be due to the difference in types or harvest times of foods. As a point of reference, the PQQ contents in foods measured by our enzymatic method were closer to that of the previously reported LC-MS/MS method [24] than the GC-MS method [23].

**Table 1:** Concentration of PQQ in food samples determined by the enzymatic method.

Sample	PQQ contents (ng/g fresh weight or ng/mL) measured by the enzymatic method
Green Tea	< 0.1
Oolong tea	< 0.1
Beer	0.77 ± 0.17
Fermented soybeans	N.D.
Green pepper	0.47 ± 0.03
Parsley	< 0.1
Rape blossoms	< 0.1
Spinach	< 0.1
Tomato	N.D.
Vinegar A	< 0.1
Vinegar B	0.23 ± 0.02
Vinegar C	0.20 ± 0.00
Vinegar D	0.80 ± 0.04
Vinegar E	2.63 ± 0.15
Vinegar F	2.48 ± 0.10
Vinegar G	3.25 ± 0.22
Vinegar H	5.41 ± 0.42

Means ± SD (n = 3), N.D. = Not detected.

Detailed analytical procedures are described in Materials and methods section.

**Fig 5:**
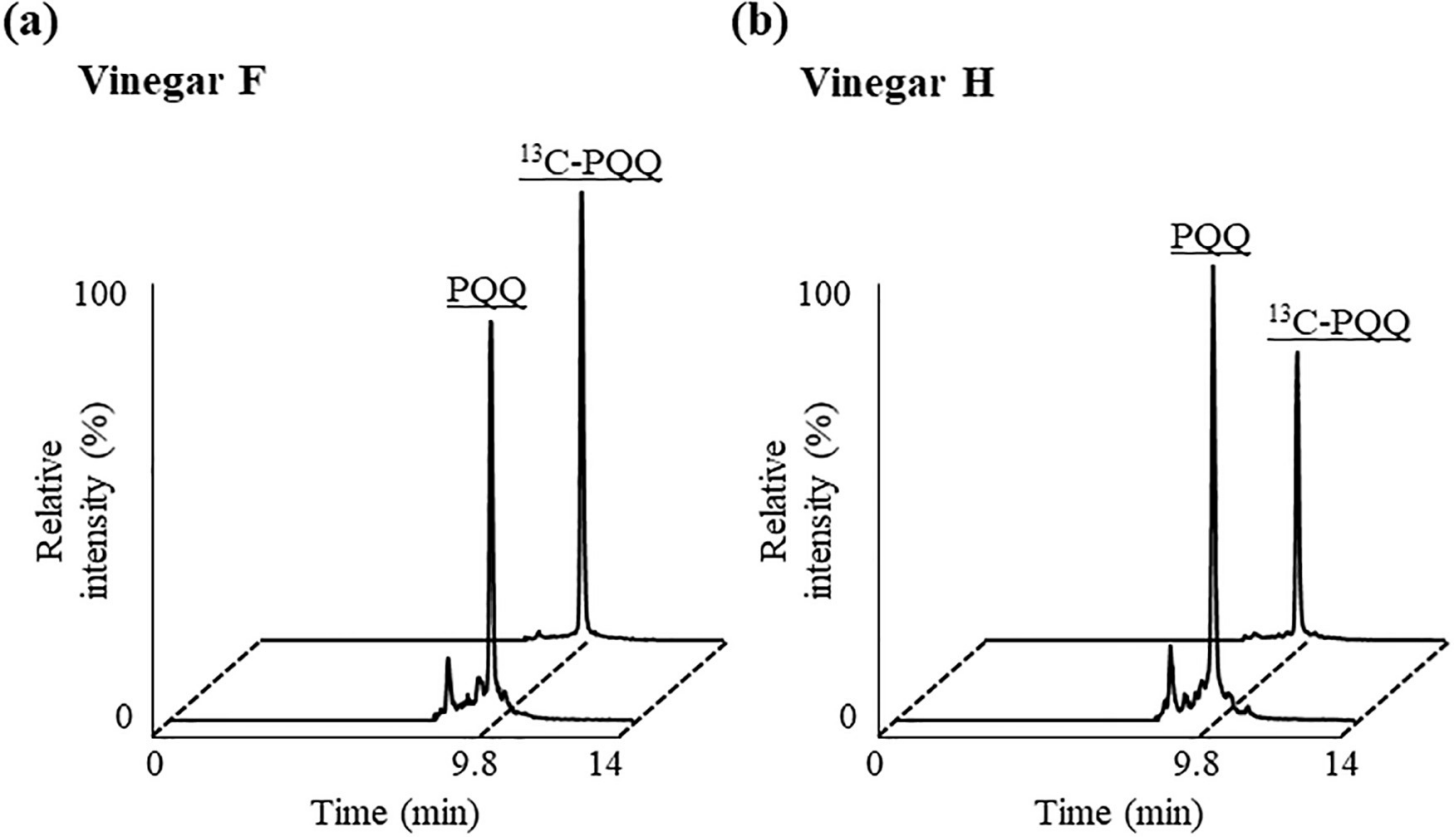
Chromatogram peaks PQQ and ^13^C-PQQ (internal standard) from extraction of vinegar F (a) and H (b). Detailed analytical procedures are described in Materials and methods section.

In addition to the foods that were previously reported to contain PQQ, with the use of the enzymatic method, we conducted a screening of foods related to acetic acid bacteria, because previous studies have shown that acetic acid bacteria possess PQQ biosynthetic genes [39, 40]. As a result, some of the foods that we conducted screening of contained high amounts of PQQ. The concentrations of PQQ in Vinegar E–H were 1.94~5.59 ng/mL and more than three-fold higher than beer which has previously been reported to contain relatively high amounts of PQQ [24]. Since it was identified by the enzymatic method that vinegar F and H contained especially high amounts of PQQ, we then tried to confirm the PQQ contents in these foods with the use of the LC-MS/MS method (Fig 5a and 5b). Consequently, the PQQ contents in the vinegar F and H were determined as 2.45 ± 0.06 ng/mL, and 4.34 ± 0.15 ng/mL by the LC-MS/MS method. Clear peaks corresponding to PQQ were also identified, and hence confirmed that these foods certainly contain a three-fold higher amount of PQQ compared to beer.

Given the above result, it was suggested that these methods were effective for screening of foods. The LOD and LOQ of the enzymatic method were more sensitive compared with LC-MS/MS analysis, and the enzymatic method additionally allowed for the simultaneous analysis of multiple samples. Also, given that the PQQ concentrations in medium determined by the enzymatic and LC-MS/MS methods were similar, we considered that both methods allow for the selective detection of only PQQ. As such, for the screening of PQQ in foods, the enzymatic method was considered more beneficial than LC-MS/MS analysis. On the other hand, LC-MS/MS MRM analysis was able to recognize PQQ specific structure and enabled to use [U-^13^]C-PQQ as an internal standard. Moreover, the PQQ values in medium and vinegar determined by LC-MS/MS analysis demonstrated smaller values of the coefficient of variation than that of the enzymatic method. Therefore, LC-MS/MS analysis could be considered more beneficial for precise quantification. Also, the LC-MS/MS method can allow for the determination of not only PQQ but also PQQ amino acid derivatives (formed by reaction of PQQ and amino acids). In order to further elucidate the distribution of PQQ in foods, simultaneous analysis of such PQQ derivatives may be necessary, and thereby LC-MS/MS analysis may be useful to conduct such analysis in future studies. Moreover, adapting the developed method to the analysis of biological samples may further enable the clarification of the absorption, metabolism, and physiological effects of PQQ.

## Conclusion

In this study, enzymatic and LC-MS/MS methods were optimized to achieve an accurate analysis of PQQ in foods. By measuring the PQQ content in various foods with two methods, we were able to identify that certain foods surely contain PQQ. The optimized methods were also applied to the screening of foods containing large amounts of PQQ. As a result, we were able to confirm that some foods related to acetic acid bacteria contain PQQ at relatively high concentrations. As mentioned above, PQQ supplementation has received attention as a possible treatment for certain diseases including dementia and diabetes. Therefore, these foods with high content of PQQ may further be applied as functional foods. Moreover, the methods optimized in this study may be applied to clarify the absorption, metabolism, and physiological effects of PQQ.

## Supporting information

S1 Fig The measurement date of the medium and foods. (XLSX)
